# Extrapolar climate reversal during the last deglaciation

**DOI:** 10.1038/s41598-017-07721-8

**Published:** 2017-08-02

**Authors:** Yemane Asmerom, Victor J. Polyak, Matthew S. Lachniet

**Affiliations:** 1 Earth & Planetary Sciences, University of New Mexico, Albuquerque, New Mexico 87131 USA; 2 Department of Geoscience, University of Nevada, Las Vegas, Las Vegas, NV 89154 USA

## Abstract

Large ocean-atmosphere and hydroclimate changes occurred during the last deglaciation, although the interplay between these changes remains ambiguous. Here, we present a speleothem-based high resolution record of Northern Hemisphere atmospheric temperature driven polar jet variability, which matches the Greenland ice core records for the most of the last glacial period, except during the last deglaciation. Our data, combined with data from across the globe, show a dramatic climate reversal during the last deglaciation, which we refer to as the Extrapolar Climate Reversal (ECR). This is the most prominent feature in most tropical and subtropical hydroclimate proxies. The initiation of the ECR coincides with the rapid rise in CO_2_, in part attributed to upwelling in the Southern Ocean and the near collapse of the Atlantic Meridional Overturning Circulation. We attribute the ECR to upwelling of cold deep waters from the Southern Ocean. This is supported by a variety of proxies showing the incursion of deep Southern Ocean waters into the tropics and subtropics. Regional climate variability across the extropolar regions during the interval previously referred to as the “Mystery Interval” can now be explained in the context of the ECR event.

## Introduction

The last deglaciation is characterized by large changes in SST^1–3^ ocean circulation^4^ and atmospheric CO_2_^5, 6^ and regional hydroclimate^7–10^. Understanding the causal interplay between these changes, is vital for attribution of large-scale climate variability. The period following the Last Glacial Maximum (LGM) may be most accessible for detailed study because of the substantial number of proxies for upwelling^6, 11–13^, changes in CO_2_ concentration and temperature. Such records are primarily from ice cores and marine proxies and are linked to a period of dramatic change in tropical and subtropical hydroclimate.

**Figure 1:**
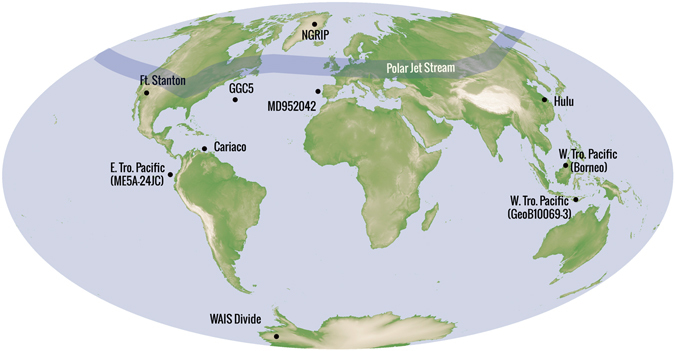
Geographic distribution of Northern Hemisphere climate proxies compared in this study and the schematic of the polar jet stream. Fort Stanton (FS-AH1), New Mexico USA (this study), NGRIP ice core record^5^, marine core MD95-2042 from the Iberian margin^25, 26^, West Antarctic Ice Sheet (WAIS) Divide ice core^22^, Bermuda Rise marine core GGC5^4^, Hulu (speleothem record)^10^, Cariaco Basin marine record^9^, Borneo speleothem record^8^, eastern tropical Pacific marine core ME0005A-24JC^2^, western tropical Pacific marine core GeoB10069-3^34^. Base map: NASA high-resolution topographic map of the earth from Shuttle Radar Topography Mission (SRTM) radar data (https://earthobservatory.nasa.gov/Features/ShuttleRetrospective/page6.php). Proxy locations done using Adobe Illustrator v. CC (http://www.adobe.com/products/illustrator.html).

**Figure 2:**
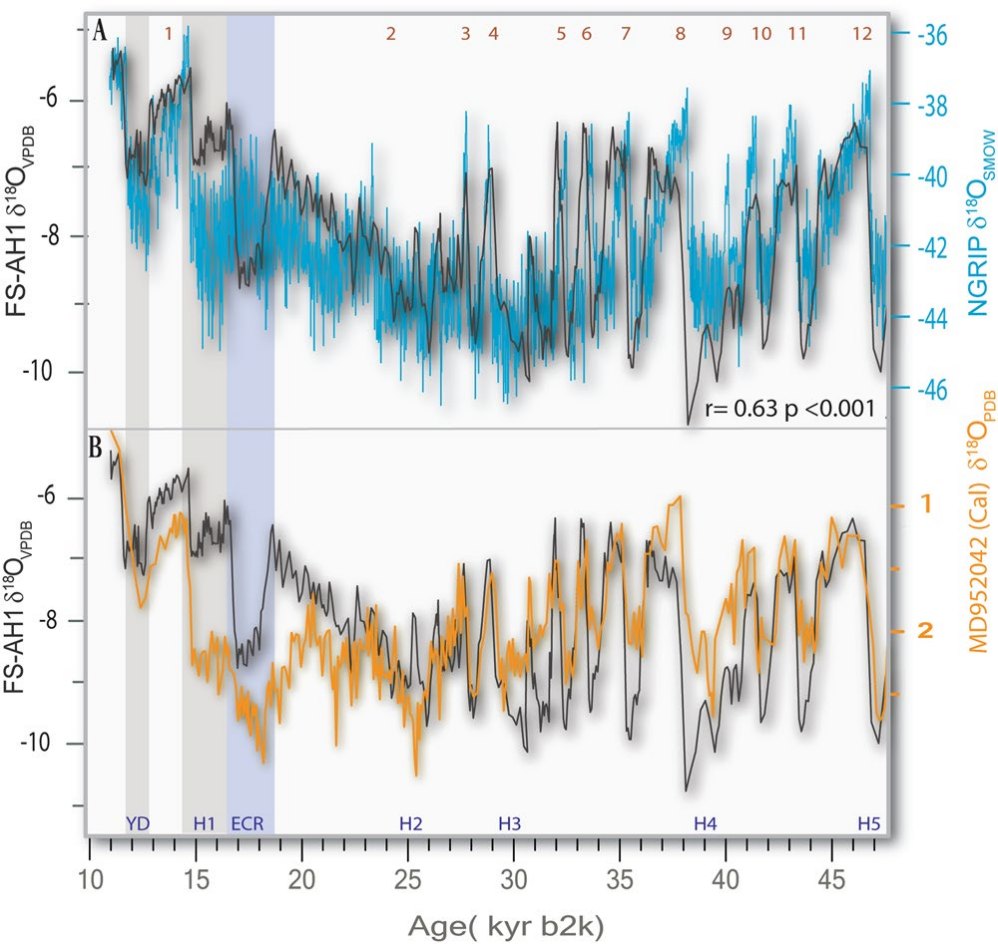
(**A**) Comparison of stalagmite FS–AH1 δ^18^O time-series (black line) and the Greenland ice-core NGRIP δ^18^O time-series on GICC05 chronology^5^. There is a remarkable match between the FS–AH1 δ^18^O time-series and ice core δ^18^O time-series, both with respect to long-term trends and millennial-scale rapid (FS-AH1 δ^18^O and NGRIP is r = 0.63, p_2t_ < 0.001) A distinct cold reversal, which we are calling the Extrapolar Climate Reversal (ECR) is shown as blue bar. (**B**) The marine core MD95-2042 δ^18^O time-series from the Iberian margin^25, 26^ tuned to the FS- AH-1 chronology (Fig. S4).

Here we present a new δ^18^O time-series from a fast-growing, precisely dated stalagmite (FS-AH1), from Fort Stanton cave, New Mexico, USA (Figs 1 and 2A, Fig. S1), the same site from which stalagmite FS-2 was collected for our earlier work showing changes in the contribution of winter precipitation, driven by changes in the position of the polar jet stream^7^. The new δ^18^O time-series (Fig. 2A) is anchored by high-precision uranium (U)-series chronology (Fig. S2) facilitated by (1) the uninterrupted high-growth rate of stalagmite FS-AH1 (triple the rate of stalagmite FS-2), (2) new gains in efficiency in multi-collector inductively coupled mass spectrometry (MC-ICPMS Neptune Plus), (3) availability of new high-purity ^233^U-^236^U spikes and, (4) more precise determinations of the half-lives of ^234^U and ^230^Th^14^.

Stalagmite FS-AH1 was collected ~1.5 km into Fort Stanton Cave (Fig. S1), where current cave temperature is stable at 10 ± 0.55 °C and relative humidity is near 100% perennially. We measured 45 U -series ages reported as years before 2000 AD (yr b2k) (Table S1) using minimal amount of powder (40–110 mg) to reduce errors related to drill-hole sizes. Our chronology shows that stalagmite FS-AH1 grew continuously from 48.4 ± 0.1 to 11.2 ± 0.1 kyr b2k. A robust age model with fully propagated errors (Fig. S2) was constructed using the program COPRA, that utilizes a Monte Carlo simulation and a translation procedure that allows for calculation of proxy time- series age uncertainties from radiometric date uncertainties^15^. The mean error across the full age range, back to 48 kyr b2k, is ± 71 years (2−σ). Coupled with the high-precision chronology, we made 422 δ^18^O measurements. The δ^18^O data have a large range, from −10.8‰ to −5.3‰ (VPDB), and are within the range of values expected for equilibrium fractionation for summer and winter precipitation (see below). The lack of δ^13^C/δ^18^O covariation (R ~ 0.00) also supports minimal kinetic isotopic effects (Fig. S3).

The imprint of millennial-scale climate variability over the last glacial period was documented in the southwestern United States by abrupt and large changes in stalagmite δ^18^O, which were previously shown to reflect, in part, changes in the ratio of winter to summer precipitation forced by meridional shifts in the polar jet stream^7, 16^. The region has two rainy seasons, consisting of summer North American monsoon rainfall, derived from the Gulf of Mexico and Gulf of California, with δ^18^O in the range of −3‰, and Pacific-derived winter precipitation with low δ^18^O, in the range of −11‰^17^. In addition to changes in the balance between winter and summer moisture, a fraction of the large range in precipitation and speleothem δ^18^O is attributed to temperature variability^7, 17^, estimated to be 5–6 °C for the region^18^ from the LGM to the Holocene. The balance between winter and summer precipitation contribution to cave infiltration is modulated by changes in the meridional position of the Northern Hemisphere Polar Jet^19^. Concurrent changes in the Asian monsoon but with the opposite effect on δ^18^O was explained as reflecting changes in position of the intertropical convergence zone (ITCZ)^7^. Alternatively, it was also suggested that ITCZ displacement during North Atlantic stadials, leading to strengthening of winter time North Pacific subtropical jet, can help explain the increase in winter precipitation in the western United States^20^. Either way, the changes are tied to Northern Hemisphere temperature variability^21^.

The salient feature of our record is that it provides a good match of both: 1) the Heinrich Stadial (HS) and Dansgaard-Oeschger (DO) events including their relative magnitudes, and 2) the secular background climate variability expressed in the NGRIP δ^18^O time-series on the GICC05 chronology^22^ (r = 0.63 p < 0.001, Fig. 2A). The two δ^18^O time-series seem linearly scalable, and the relative amplitude match is remarkable for most of the record. Whether the Greenland ice-core δ^18^O temperature proxy record represents a hemispherical-scale record of temperature variability is still an open question, because some studies have suggested that it may contain significant imprints of local temperature^23^. Moreover, ice-core and speleothem δ^18^O are an imperfect temperature proxy because they are also strongly influenced by complex moisture source and air mass histories^24^. The one-to-one match between stalagmite FS-AH1 and NGRIP δ^18^O for DO events, including the sharp stadial to interstadial transitions (Fig. 2A), as well as the long-term trends indicates﻿ that the two proxies represent hemispherical scale climate variability.

The long-term coherency of the coupling between North Atlantic sea surface temperature (SST) and changes in northern hemisphere atmospheric temperature are tested by comparing the FS-AH1 δ^18^O time-series (Fig. 2A) to the long marine core MD95-2042 δ^18^O time-series from the Iberian margin (Fig. 2B)^25, 26^, which was the basis for a North Atlantic SST reconstruction^1^. Because of the inherent problems with ^14^C dating of marine sediments, we tuned the MD95-2042 chronology to our speleothem chronology by matching some of the inflection points of the HS and DO events (red dots in Fig. S4), while not changing the relative age differences of data points occurring between the tune points in order not to change the topology of the overall record (see Supplemental Information). As a result, we did not modify peak shapes. The remarkable match between the two records (Fig. 2A,B) is similar to the match between the Greenland ice-core and stalagmite FS-AH1 data (Fig. 2A). The combined speleothem, Greenland ice and marine δ^18^O data demonstrate coherent atmospheric temperature and ocean circulation coupling during the Last Glacial, particularly during the sharp DO transitions (Fig. 2A).

**Figure 3:**
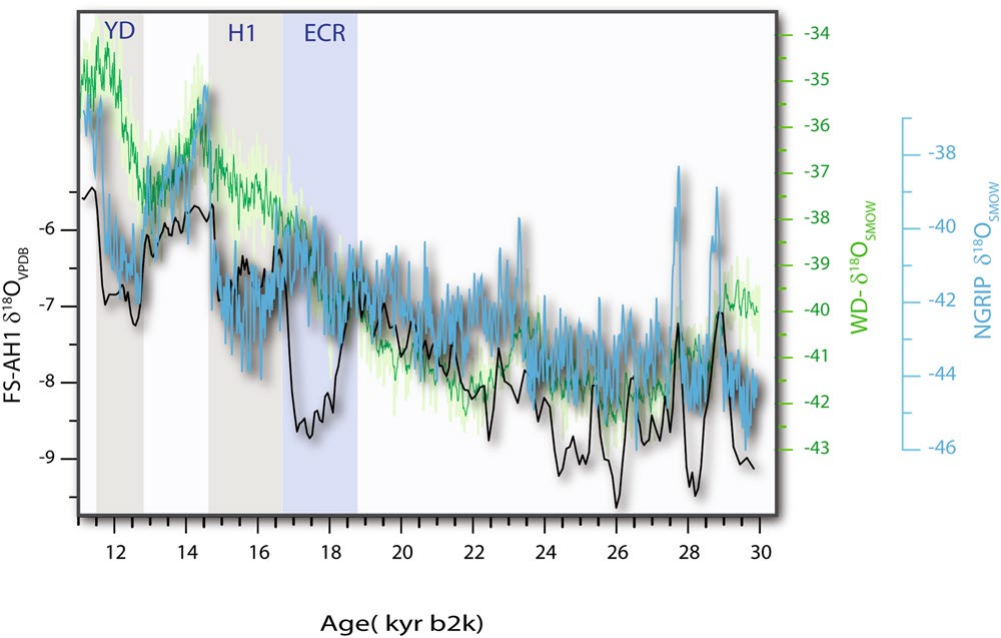
Detailed comparison of the Northern Hemisphere Greenland ice-core NGRIP δ^18^O time-series^5^ and the Southern Hemisphere polar ice core West Antarctic Ice Sheet (WAIS) Divide ice core δ^18^O data (WDC)^22^ against the FS- AH1 record. The secular trends in the three records are remarkably similar through the LGM, showing that the growth of Northern Hemisphere ice sheets and deglaciation took place in the background of concurrent bi-polar warming with only a slight change during the ECR in both poles. The two poles diverge during the Northern Hemisphere cooling associated with HS1 proper and the three records rapidly changing during the transition to the Bølling-Allerød. The largest shift in the AH1 occurs during ECR, which is also the case for many tropical and subtropical hydroclimate and SST proxies (Fig. 6).

The only pronounced stadial in FS-AH1 and North Atlantic sediment cores that is not clearly expressed in the Greenland ice core record is herein referred to as the Extrapolar Climate Reversal (ECR) starting at 18.6 kyr b2k (Figs 2A and 3) and lasting for a little over a thousand years, to the beginning of HS-1. The FS-AH1 data fully resolve HS-1 from the ECR, which underscores the fact that the FS-AH1 is closely tied Greenland, except for the ECR. This anomaly also is missing from West Antarctic Ice Sheet (WAIS) Divide ice core δ^18^O time-series (WDC) (Fig. 3) that is thought to reflect coupled ocean-atmosphere temperature variability^27^ and thus the ECR interpreted to be extra-polar.

**Figure 4:**
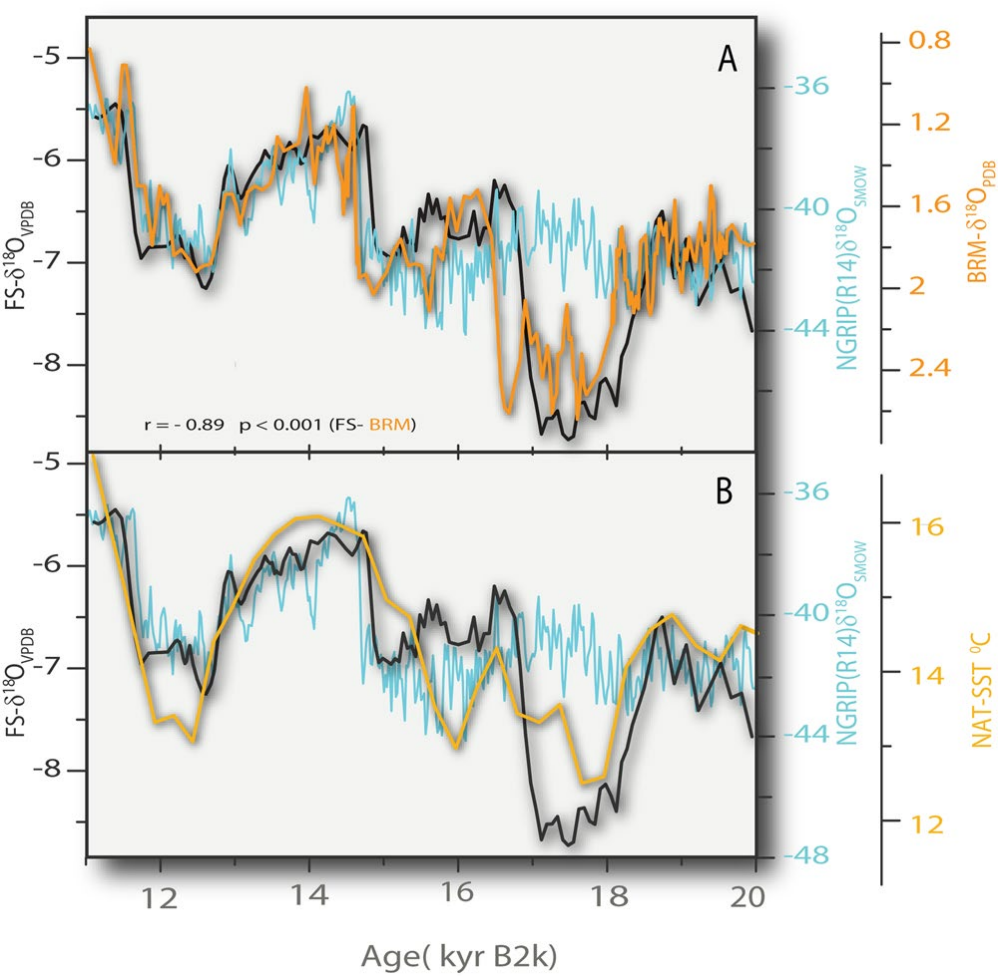
(**A**) A comparison between FS-AH1 δ^18^O data (FS; black curve), the Bermuda Rise - δ^18^O data (orange line)^4^ and NGRIP δ^18^O time-series (blue curve)^5^. The FS-AH1 and the Bermuda Rise δ^18^O data show remarkable correlation (r = −0.89 *p* < 0.001) during the period shown here, covering the ECR and HS1, associated with a weakened AMOC^4^. (**B**) Comparison of FS-AH1 δ^18^O data to another North Atlantic δ^18^O record, MD952042 off the Iberian margin (yellow curve)^1^ showing the similarity between the FS-AH1 δ^18^O and and the SST reconstruction for the North Atlantic during the period of the ECR and HS1^1^.

**Figure 5:**
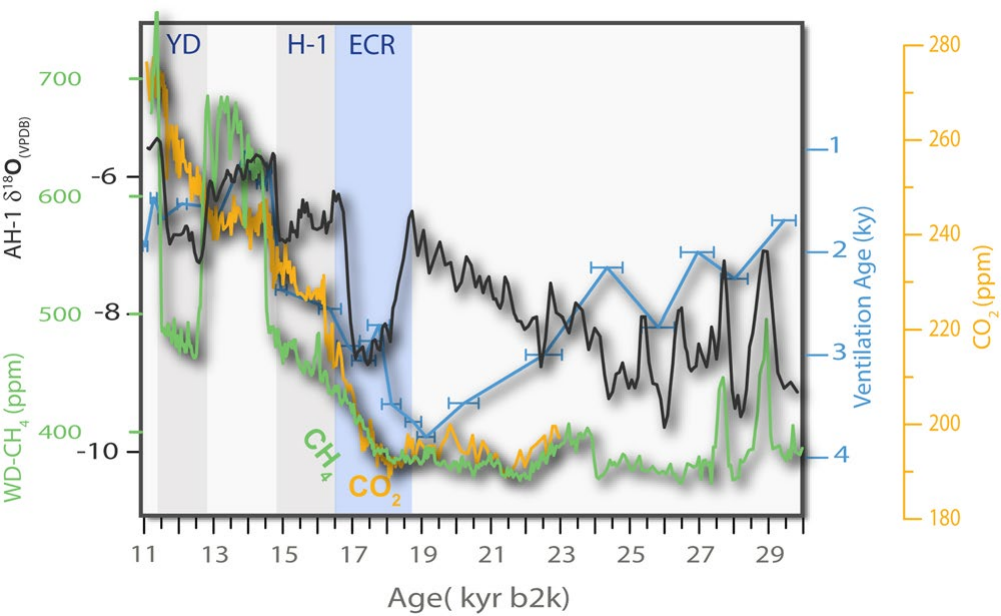
Variations in the Δ^14^C -derived ventilation ages^6^ (blue line) compared to FS-AH1 δ^18^O and changes in the concentration of atmospheric CO_2_^27^ (yellow line). The timing of the steep decrease in ventilation ages, presumed to result from upwelling in the Southern Ocean sector of the AMOC coincides with the timing of the ECR and the rise in atmospheric CO_2_. Upwelling of cold Southern Ocean water and flooding of the subtropics and the tropics may have led to differential cooling of the subtropics and the tropics, while the secular trends in the polar regions reflect the combined effects of their summer insolation.

The cause of large-scale hydrologic variability in the interval encompassing the ECR and HS1 coincides with the time of AMOC collapse^4^ (Fig. 4A) and a dramatic reduction in subtropical Atlantic SST^1^ (Fig. 4B). The beginning of the ECR is synchronous with the initiation of atmospheric CO_2_ rise^22^, and the steep decrease in the ventilation ages of deep water reservoirs of the Southern Ocean, derived from the offset between benthic foraminifera and atmospheric ^14^C ages^6^, both attributed to increased upwelling in the Southern Ocean (Fig. 5). The excursion of deep waters from the Southern Ocean are expressed very distally, for example in the north Pacific Ocean, based both on Δ^14^C^12^ and Nd^11^ isotopic data and the North Atlantic ocean, based on silica concentration data^13^. All these proxy data support continuous incursion of cold deep, Δ^14^C- depleted Southern Ocean waters in to the extrapolar oceans during the period of the ECR event. More directly, a potential teleconnection between Southern Ocean upwelling and changes in the tropical Pacific hydrology is suggested thorough changes in the flux of Antarctic Intermediate Water (AAIW)^11^. Changes in the SST gradients between the northern eastern tropical Pacific and southern eastern tropical Pacific^3^ follow the changes seen in our FS-AH1 record, the lowest gradient coinciding with the tropical and subtropical hydrology described below.

**Figure 6:**
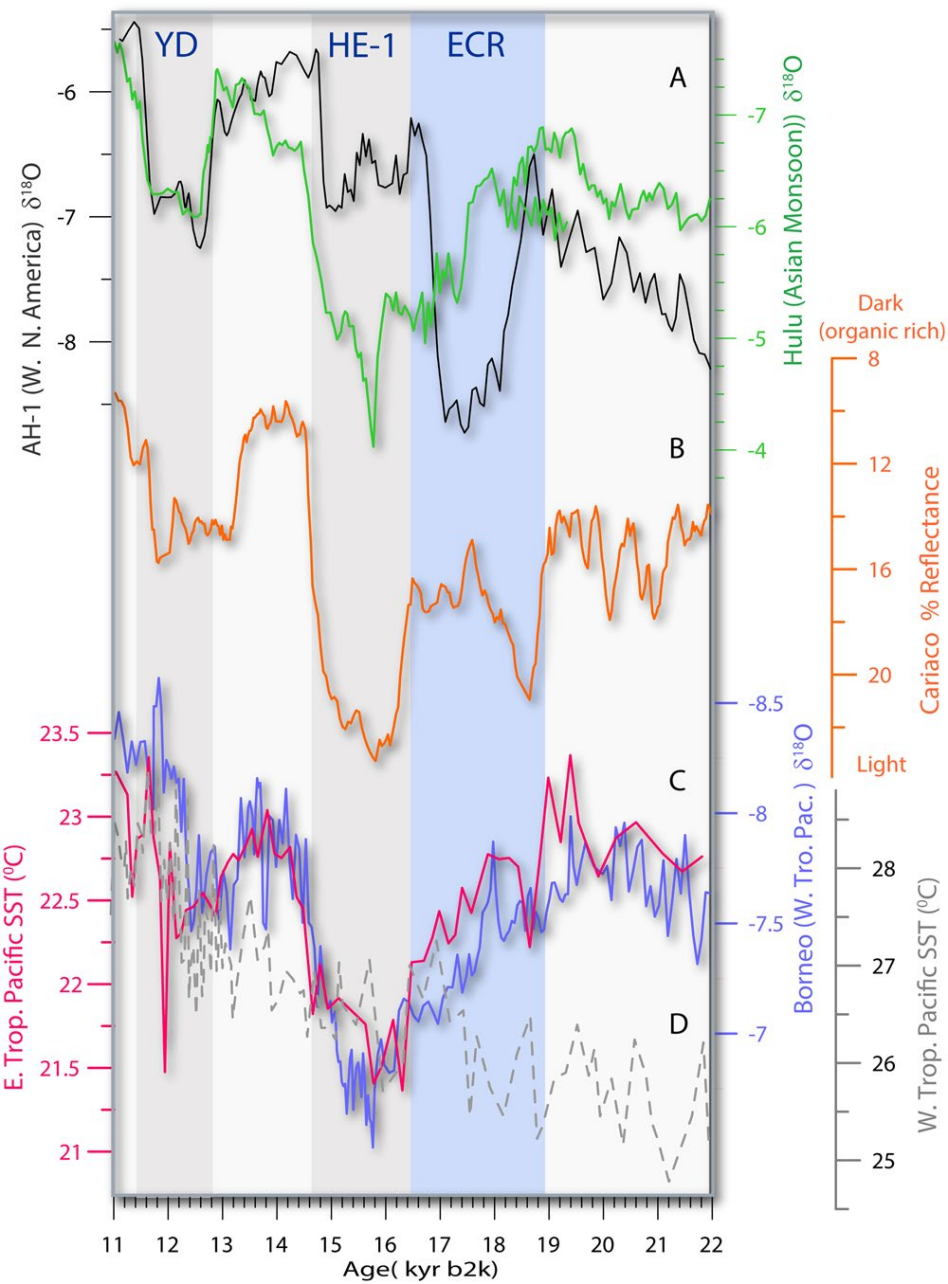
(**A**) The ECR is expressed well in the East Asian Hulu speleothem monsoon record^10^ (green composite line) as increasing δ^18^O values indicating a weaker monsoon. (**B**) Cariaco Basin reflectance data are shown as orange line plotted against FS- AH-1 temperature proxy data^9^. Interstadials (strong AMOC) are characterized by low reflectance (high organic content) layers, while stadials (weaker AMOC) are marked by high reflectance (low organic matter, oxidized layers). The ECR and HS1 are separated in both the Cariaco Basin and the FS- AH-1 records because of their strong link to the meridional modulation by the AMOC and coupled ocean-atmosphere variability (Fig. 2A,B). (**C**) The same trend is observed in the western tropical Pacific convection speleothem record (gray line)^8^, whereby weaker convection associated with the ECR event is indicate sever weakening of western tropical Pacific moisture and notable synchronous changes in eastern tropical Pacific SST^2^. A western tropical Pacific SST is shown in gray^34^.

The fingerprint of the 18.6 kyr b2k climate transition is expressed prominently in many records around the globe, primarily in subtropical and tropical climate regimes (Fig. 6A,B), such the SE Asian monsoon expressed in the Hulu record^10^, the Cariaco tropical marine record^9^ and the concurrent weakening of tropical Pacific convection^8^ (Fig. 6A). The hydroclimate changes during the ECR is the most prominent feature in most subtropical and tropical climate records. Lowering of tropical and subtropical SSTs^1^ (Fig. 4B) leading to decrease in net equatorial heat transport, with the slowdown of the AMOC^4^ (Fig. 4A) would weaken the ITCZ^28^ resulting in southward shift of the Asian Monsoon^10^ (Fig. 6A). Similarly southward displacement of the ITCZ during the ECR, would lead to strengthening of winter time North Pacific subtropical jet, and increased winter precipitation in the western United States^20^.

Although we showed that stalagmite FS-AH1 δ^18^O variability is coupled to changes in the meridional position of the polar jet stream^7^, the hydroclimatic response in extrapolar regions may also be linked to changes in the ITCZ position, because such variations appear to be decoupled from Greenland and Antarctic temperatures during this interval. It has been previously suggested that variability in the proportion of winter versus summer precipitation in the southwest USA, reflected by the stalagmite δ^18^O record, may be explained by changes in the position of the ITCZ^20^. The Cariaco record most resembles the FS-AH1 record to the extent that the ECR and HS1 are clearly resolved in both records due to their direct link to the AMOC^9^ (Fig. 6B). But there is also a difference in detail. The Cariaco record shows a rise in organic carbon content after initial rapid decrease, followed by another decrease during HS1 (Fig. 6B). The rise in organic carbon may reflect the infusion of nutrient-rich water due to the upwelling.

The period covering the ECR and HS1 is expressed prominently by changes in tropical hydrology, including the collapse of western tropical Pacific rainfall^8^ (Fig. 6C) and changes in SST in the eastern tropical Pacific^2^, and narrowing of the gradient across it^3^. Although we suggest that the ECR and HS1 are two distinct events with different causes, the impact of these two events is similar across a range of tropical and subtropical regions and are most likely driven by changes in the long-term position of the ITCZ. Both models and proxy data in general^29, 30^ and for the western tropical Pacific in particular^31, 32^ show a southward shift of the mean ITCZ position during cold events and drier than normal conditions^32^, consistent with the patterns observed in western tropical Pacific rainfall (Fig. 6C).

Our analysis of proxy data in the context of the global SST and hydroclimate data show that Southern Ocean upwelling had a profound influence across the globe, providing a coherent framework for understanding large changes in hydroclimate during a period previously dubbed as the mystery interval^33^.

## Methods

U-series isotope measurements were made at the Radiogenic Isotope Laboratory, University of New Mexico. Subsample powders (40–110 mg) were drilled and dissolved in nitric acid and spiked with a mixed ^229^Th-^233^U-^236^U spike. U and Th were separated using conventional anion-exchange chromatography. U and Th measurements were made on a Thermo Neptune plus configured multicollector inductively coupled plasma mass spectrometer (MC-ICPMS). The MC-ICPMS measurements were run in static mode using a mix of 10^11^ and 10^12^ Ω resistors in conjunction with five Faraday cup detectors and an ion-counting secondary electron multiplier detector, following the method described in ref. 35. The CRM-145 U isotope standard was measured with the samples, obtaining the conventionally accepted δ^234^U value of −38.5^14^. U and Th procedural blanks were in the range of 5–10 pg and therefore have no effect on ages. The analytical uncertainties are 2σ of the mean. The age uncertainties include analytical errors and uncertainties in the initial ^230^Th/^232^Th ratios. Initial ^230^Th/^232^Th ratios were corrected using an empirical value of 30 ± 15 ppm, allowing for a range of contributions, from silicate to carbonate materials. The sample is very clean and thus not sensitive to initial ^230^Th correction, in addition to the fact that the samples are older than 10 kyrs.

Powders for δ^18^O analysis were sampled along the FS-AH1 growth axis at 1 mm intervals and were analyzed at the Las Vegas Isotope Science Lab (LVIS) of the University of Nevada, Las Vegas. Stalagmite powders were reacted with three drops of anhydrous phosphoric acid at 70 °C in a ThermoElectron Kiel‐IV automated carbonate preparation device coupled to a Delta V Plus mass spectrometer. Values are reported in standard permil (‰) notation with respect to Vienna Pee Dee Belemnite (VPDB). Internal standard precision is better than 0.08‰ δ^18^O.

### Data accessibility

The AH-1 U-series data are included in the Supplemental Materials; the δ^18^O data are archived at the NOAA National Centers for Environmental Information website

## Electronic supplementary material

Supplementary information

Table 1S
