# Supplementary material for: Extrapolar climate reversal during the last deglaciation
Source: Sci Rep. 2017 Aug 2;7:7157. doi: 10.1038/s41598-017-07721-8 (PMC5541005; doi:10.1038/s41598-017-07721-8)
Supplement: Supplementary file 1 — Supplementary information [file 41598_2017_7721_MOESM1_ESM.pdf]

Supplemental Information

Extrapolar climate reversal during the last deglaciation

Yemane Asmerom, Victor J. Polyak, Matthew S. Lachniet

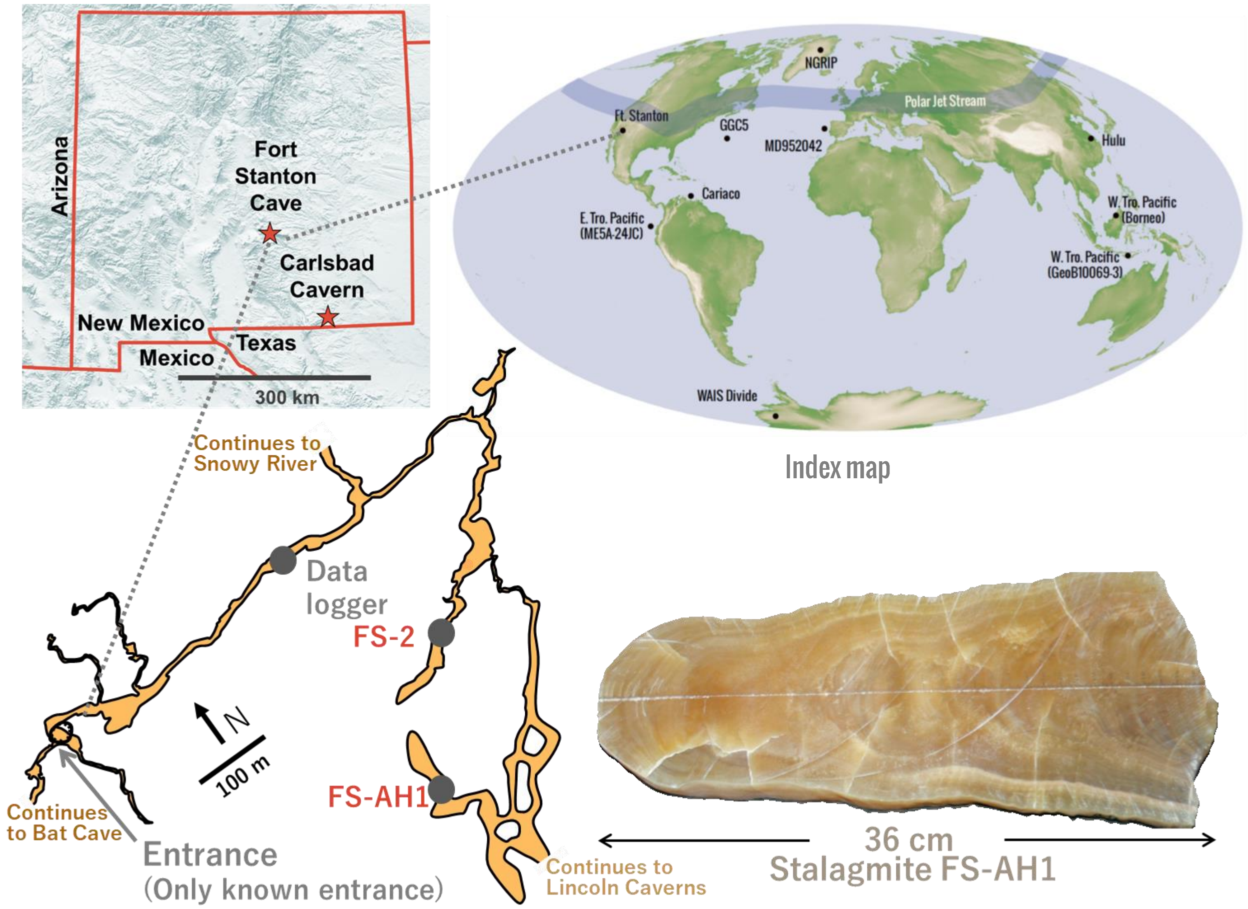

Figure 1S. General map of a portion of Fort Stanton Cave showing the only known entrance to the cave and collection sites of stalagmite FS-AH1. The cave has a stable temperature 10 ± 0.2°C and relative humidity near 100% perennially[1]. Note that Fort Stanton Cave is sensitive to Polar Jet meridional variability. [Map courtesy of Steve Peerman, Project Director and John Corcoran, cartographer, Ft. Stanton Cave Study Project]. Index map: NASA high-resolution topographic map of the earth from Shuttle Radar Topography Mission (SRTM) radar data (https://earthobservatory.nasa.gov/Features/ShuttleRetrospective/page6.php) .

Proxy locations done using Adobe Illustrator v. CC (http://www.adobe.com/products/illustrator.html)

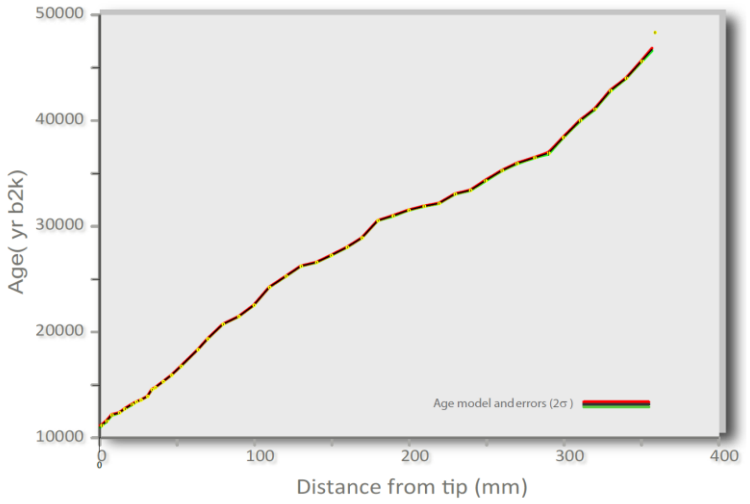

Figure 2S. U-series based age model (black line) for speleothem FS-AH1. The 2σ uncertainty bands are shown as red (upper) and green (lower) curves. The age model was constructed using the COPRA[2]. Individual samples are shown as yellow dots, with their 2σ errors, smaller than the symbol, (shown as black bars).

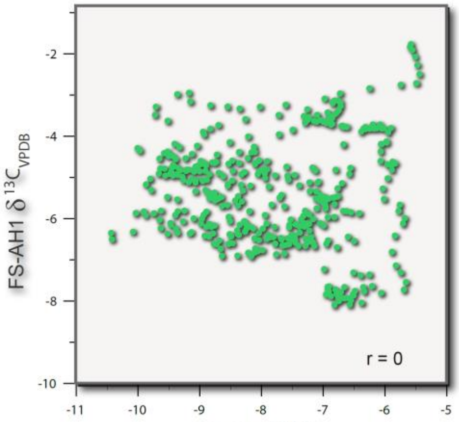

Figure 3S. A plot of $\delta^{18}O$ vs $\delta^{13}C$ data showing there is no correlation between the two data sets (r=0). This is a good indication that kinetic fractionation did not have significant effect, consistent with the fact that the sample location has 100% relative humidity and very stable cave temperature at 10 ± 0.2°C (over two years of measurement) .

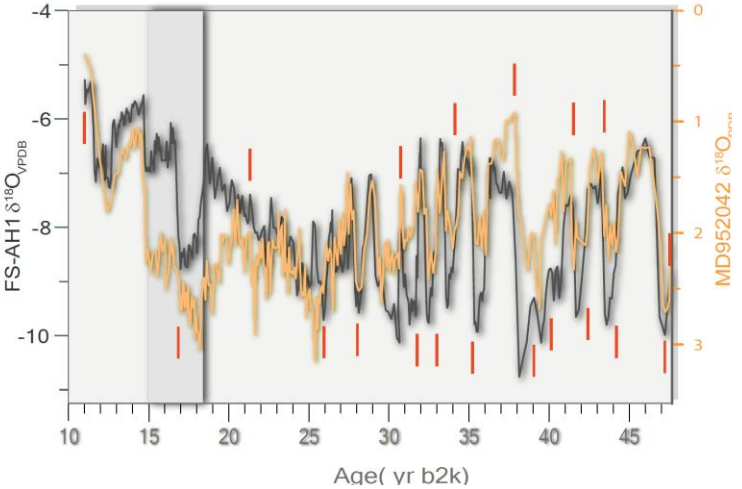

Figure 4S. The anchor points for calibrating the chronology of MD952042 marine core time-series from the Iberian margin[3,4] against the AH-1 chronology, shown as red bars. The ages of points in between the anchor points were recalculated by keeping the original relative distance unchanged, in order not to change the topology of the time series. Our approach is shown in the Methods section.

Procedure for tuning the Greenland NGRIP[3] and Iberian coast MD95-2042

We took clear common inflection points between FS-AH1 $\delta^{18}O$ chronology and corresponding inflection points in the MD95-2042 time-series[3,4] as shown in Fig. 4S and used them as anchors. The ages of points in between the anchor points were recalculated by keeping the original relative distance unchanged, in order not to change the topology of the time series. Illustrated schematically here.

Original Ages	FS-AH1 anchor ages	Tuned ages
X_a	Y_a [anchor a]	$Z_a = Y_a$
X_1		$Z_1 = Y_a + [(Y_b - Y_a)/(X_b - X_a)*(X_1 - X_a)]$
X_2		---
X_3		$Z_3 = Y_a + [(Y_b - Y_a)/(X_b - X_a)*(X_3 - X_a)]$
---		---
X_n		$Z_n = Y_a + [(Y_b - Y_a)/(X_b - X_a)*(X_n - X_a)]$
X_b	Y_b [anchor b]	$Z_b = Y_b$

Supplemental References

1 Asmerom, Y., Polyak, V. J. & Burns, S. J. Variable winter moisture in the southwestern United States linked to rapid glacial climate shifts. *Nature Geoscience* **3**, 114-117 (2010).

2 Breitenbach, S. *et al.* Constructing proxy records from age models (COPRA). *Climate of the Past* **8**, 1765-1779 (2012).

3 Cayre, O., Lancelot, Y., Vincent, E. & Hall, M. A. Paleoceanographic reconstructions from planktonic foraminifera off the Iberian Margin: temperature, salinity, and Heinrich events. *Paleoceanography* **14**, 384-396 (1999).

4 Shackleton, N. J., Hall, M. A. & Vincent, E. Phase relationships between millennial-scale events 64,000–24,000 years ago. *Paleoceanography* **15**, 565-569 (2000).
